# Innovative Low-cost Probe Generation Empowers Targeted Long-read RNA Sequencing

**DOI:** 10.1093/gpbjnl/qzae027

**Published:** 2024-04-10

**Authors:** Gang Fang

**Affiliations:** Department of Genetics and Genomic Sciences, Icahn School of Medicine at Mount Sinai, New York, NY 10029, USA

Alternative splicing of human genes is a cornerstone of genetic complexity and diversity, enabling a single gene to produce many different proteins [1]. This is not only crucial for human development but also plays pivotal roles in many diseases [2]. Over the years, next-generation sequencing (NGS) has been instrumental in unraveling the intricacies of human gene splicing [3]. It has led to significant advancements in our understanding, with notable examples being the identification of splice variants in *BRCA1* and *BRCA2* genes, which are critical in breast and ovarian cancer risk assessment [4].

However, the complexity of human genes, which consist of ten exons on average, poses a challenge for the short-read sequencing approaches commonly used in NGS platforms like Illumina. Specifically, the combinatorial patterns of tens of exons can generate many isoforms, which are very challenging or impossible for short reads (up to 300 bp) to resolve. In contrast, long-read sequencing technologies, such as those offered by Oxford Nanopore Technologies (ONT) and Pacific Biosciences, represent a paradigm shift. Their ability to read several kilobases to tens of kilobases in a single read allows them to capture entire transcripts, end-to-end [5]. This has led to groundbreaking discoveries in gene splicing, unveiling a new era of research in human health and disease [6,7]. Large-scale studies using these technologies have reported the discovery of tens of thousands of novel isoforms [8].

One significant hurdle with long-read sequencing, however, is its high cost. This is particularly problematic in isoform discovery, where some critical human genes are of low abundance, and their isoforms vary greatly in terms of abundance. Consequently, a large portion of sequencing effort and yield is often consumed by housekeeping genes or genes not of primary interest in specific studies, leading to inadequate coverage of the target genes. While deep sequencing with small sample sizes might be a workaround, this approach is not scalable for larger studies or broad clinical applications. This underscores the need for efficient enrichment methods.

Probe-based enrichment strategies have been widely used in various applications, such as exome sequencing and targeted panels for both genomic DNA and cDNA, especially in cancer research. For example, in the realm of oncology, several targeted panels have been pivotal in identifying genetic mutations across a range of cancers, facilitating personalized treatment approaches [9]. However, the high cost of probe synthesis has been a significant bottleneck in probe-based target enrichment. This challenge stems from the expenses involved in creating custom probes necessary for targeted sequencing. These costs can be prohibitive, especially when a large number of probes are needed to cover complex genes comprehensively. Specifically for targeted long-read RNA sequencing, hybridization capture-based enrichment using biotinylated capture oligos, like RNA Capture Long Seq [10], is effective but the commercially synthesized oligos are costly and limited in their number of uses, leading to high per-sample capture costs. An alternative method, ORF Capture-Seq, utilizes biotinylated oligos synthesized from ORF clones but is limited by resource-consuming access to the human ORFeome library and its applicability to only genes included in the library [11].

Addressing the cost challenges associated with probe-based methods, a recent study by a team at Children’s Hospital of Philadelphia led by Professors Yi Xing and Lan Lin introduced TEQUILA-seq [12]. The core innovation of TEQUILA-seq is its use of nicking-endonuclease-triggered isothermal strand displacement amplification. This approach significantly reduces the per-reaction cost of targeted capture by 2–3 orders of magnitude, compared to standard commercial solutions. For example, while a standard commercial approach for a 6000-probe panel might cost approximately $813 per reaction, TEQUILA-seq achieves the same for $0.31–$0.53 per reaction. This significant cost reduction is achieved without compromising the quality or efficiency of probe synthesis, as demonstrated by comprehensive evaluation relative to xGen Lockdown (IDT) probe-based target enrichment and sequencing. Furthermore, TEQUILA-seq not only matches but also enhances the detection of target transcripts while preserving their quantification, as demonstrated by the evaluation with neuroblastoma cells mixed with synthetic transcripts including External RNA Controls Consortium (ERCC) standards and Spike-In RNA Variants (SIRVs).

To demonstrate the power of TEQUILA-seq in real applications, the team performed an in-depth study profiling full-length transcript isoforms of 468 actionable cancer genes. Applied across a diverse array of 40 representative breast cancer cell lines, TEQUILA-seq identified novel transcript isoforms in critical tumor suppressor genes (TSGs) like *TP53*. In addition, TEQUILA-seq discovered 54 breast cancer subtype-associated transcript isoforms in 50 genes, highlighting the method’s precision in detecting subtle yet crucial differences in gene expression across various cancer subtypes. From these rich data, the study also highlighted a common mechanism of TSG inactivation in cancer cells. Specifically, the analysis showed that TSGs are significantly more enriched for aberrant transcript isoforms targeted for degradation via mRNA nonsense-mediated decay (NMD), offering new insights into the molecular mechanisms underlying cancer development and progression.

Building on the initial success of TEQUILA-seq, the study further performed several technical refinements. Firstly, the probe synthesis yield was significantly improved by varying the amounts of template oligo pool and incubation time. Higher template amounts and longer incubation periods up to 8 h resulted in increased probe yields, a trend that plateaued likely due to enzyme and dNTP exhaustion. Secondly, the introduction of blocking oligos targeting various elements of the probe synthesis and cDNA amplification process led to modest improvements in capture performance, enhancing on-target rates by 2.8% to 5.6%. Finally, the implementation of multiplexing using the native barcoding kit for the R10.4 nanopore sequencing chemistry allowed efficient and accurate multiplexed TEQUILA-seq on four breast cancer cell lines, generating 7.2 million reads with an impressive 88.4% unique assignment rate to the correct cell lines. This enhancement not only scaled up the process and reduced costs but also demonstrated the potential of TEQUILA-seq for use with higher-throughput long-read sequencers like the ONT PromethION platform, ensuring its applicability in a wider range of research settings.

TEQUILA-seq, with its versatile and cost-effective approach, holds potential for various applications in targeted long-read RNA sequencing. Originally demonstrated as a proof-of-concept for profiling actionable cancer genes, its scope extends far beyond, capable of being tailored to any gene panel for specific transcript isoform discovery and quantification. For instance, it can be pivotal in RNA-guided genetic diagnosis of Mendelian disorders and in identifying oncogenic gene fusions for precision oncology. While primarily designed for poly(A)+ RNAs, TEQUILA-seq can also adapt to non-poly(A)+ RNAs through minor protocol adjustments. An important aspect of TEQUILA-seq is its compatibility with both long-read and short-read RNA sequencing workflows, making it a versatile tool in genomic research. Beyond RNA sequencing, TEQUILA probes also offer possibilities in targeted DNA sequencing applications, including DNA methylation analysis and chromatin conformation studies.

TEQUILA-seq is expected to be broadly applicable for several reasons. (1) TEQUILA-seq lowers research barriers. The cost-effective nature of TEQUILA-seq dramatically lowers the financial barriers for researchers exploring targeted long-read RNA sequencing to study gene splicing. Traditionally, the high cost of designing and synthesizing probes, especially for genes with complex splicing patterns, has been a significant hurdle. TEQUILA-seq’s approach not only makes it more affordable to design a greater number of probes for comprehensive gene coverage but also allows for deeper sequencing. This facilitates a more detailed and accurate analysis of splicing patterns, which is crucial for understanding the underlying mechanisms of various genetic disorders and developmental processes. (2) TEQUILA-seq enables efficient resource allocation. By enhancing the efficiency of long-read sequencing throughput, TEQUILA-seq ensures that resources are more effectively allocated. This is particularly relevant in comparative studies, such as those contrasting cancerous and normal cells, where detailed and accurate sequencing data are paramount. The ability to focus sequencing efforts more precisely means that researchers can generate high-quality data with fewer resources, enabling broader and more detailed studies with the same or lower budget. (3) TEQUILA-seq boosts research impact. The widespread adoption of TEQUILA-seq has the potential to significantly accelerate genomic research. By making targeted long-read RNA sequencing more accessible, it opens the door for a wider range of scientists to contribute to this field. This democratization of advanced sequencing technologies could lead to a surge in discoveries, particularly in understanding complex genetic diseases and developing novel therapeutic approaches.

In conclusion, TEQUILA-seq represents a significant advancement in genomics, offering a cost-effective, efficient, and versatile method for targeted long-read sequencing. Its impact extends from academic research to clinical applications, promising to broaden the scope of genetic research and make precision medicine more accessible and effective. The implications of this technology are vast, setting the stage for groundbreaking discoveries and innovations in the understanding and treatment of a diversity of genetic disorders.

## CRediT author statement

**Gang Fang:** Conceptualization, Writing – original draft, Writing – review & editing. The author has read and approved the final manuscript.

## Competing interests

The author has declared no competing interests.
